# Improving Pathways for Patients With Disordered Eating in General Acute Hospital, in Accordance With MEED Guidelines

**DOI:** 10.1192/bjo.2024.537

**Published:** 2024-08-01

**Authors:** Alisha Bakshi, Kushal Varma, Emma Barrow, Adelle Williams, Marcus Mottershead

**Affiliations:** 1 University Hospitals Birmingham NHS Foundation Trust, Birmingham, United Kingdom; 2 Birmingham and Solihull Mental Health Foundation Trust, Birmingham, United Kingdom

## Abstract

**Aims:**

Patients with disordered eating in psychiatry are considered highly complex in the acute hospital setting. In Spring 2023 a pilot for a specialised dietitian was introduced to identify and target such patients; aimed at reducing length of stay to the acute medical wards. Hospital admissions for eating disorder increased by 84% between 2015/16 and 2020/21; with increasing complexity of presentations and a demand for Specialist Eating Disorder (SEDU) beds, there are increasing numbers admitted to acute medical beds for initial treatment and management. In 2021 the Royal College of Psychiatrists published its updated guidance, Medical Emergencies in Eating Disorders (MEED). There is recognition that acute trusts must identify care pathways for the management of patients with eating disorders and severe food restriction for psychiatric reasons. This audit aims to show how these guidelines are being implemented locally and where there is a need for improvements in care pathways focusing particularly on length of stay, frequent attenders and avoiding hospital admissions.

**Methods:**

A retrospective audit of 26 patients presenting between 01/03/2023 and 31/12/2023 was completed. Patients were identified from data collated by the specialist dietitian as having presented with an existing diagnosis of eating disorder or disordered eating in the context of psychiatry. Some patients were detained under the Mental Health Act. Some patients presented on multiple occasions to the acute hospital during this period; each inpatient episode was analysed independently. Data was collected retrospectively by analysing PICS documentation (electronic notes system) and entered into a data collection spreadsheet. A Google Form checklist was created to capture whether key points from MEED guidelines were met.

**Results:**

Demographic data, details of initial presentation and admission events were collated including the team initially referred to and how long after the initial admission this occurred. Outcomes of admission were also recorded. Data was quantitatively analysed to understands trends in referral process, MDT working (inclusion of emergency clinicians, acute medicine, psychiatrist, specialist dieticians and nursing colleagues). Average lengths of stay, number of attendances and planned admissions were also captured.

**Conclusion:**

An overall reduction in length of stay for detained patients with dietetic and wider MDT input was noted from 50 days prior to January 2023, to 29 in the period from March 2023 onwards. Frequent attendance for electrolyte abnormalities was significantly improved though implementing MDT working with teams in the community and planned admissions from inpatient units or SEDUs for medical management reduced overall length of stay for those patients.

